# Retrospective Modeling of the Omicron Epidemic in Shanghai, China: Exploring the Timing and Performance of Control Measures

**DOI:** 10.3390/tropicalmed8010039

**Published:** 2023-01-05

**Authors:** Lishu Lou, Longyao Zhang, Jinxing Guan, Xiao Ning, Mengli Nie, Yongyue Wei, Feng Chen

**Affiliations:** 1 Department of Biostatistics, School of Public Health, Center of Global Health, Nanjing Medical University, Nanjing 211166, China; 2 Center for Public Health and Epidemic Preparedness & Response, Peking University, Xueyuan Road, Haidian District, Beijing 100191, China

**Keywords:** COVID-19, Shanghai Omicron epidemic, transmission dynamics, timing of control measures

## Abstract

Background: In late February 2022, the Omicron epidemic swept through Shanghai, and the Shanghai government responded to it by adhering to a dynamic zero-COVID strategy. In this study, we conducted a retrospective analysis of the Omicron epidemic in Shanghai to explore the timing and performance of control measures based on the eventual size and duration of the outbreak. Methods: We constructed an age-structured and vaccination-stratified SEPASHRD model by considering populations that had been detected or controlled before symptom onset. In addition, we retrospectively modeled the epidemic in Shanghai from 26 February 2022 to 31 May 2022 across four periods defined by events and interventions, on the basis of officially reported confirmed (58,084) and asymptomatic (591,346) cases. Results: According to our model fitting, there were about 785,123 positive infections, of which about 57,585 positive infections were symptomatic infections. Our counterfactual assessment found that precise control by grid management was not so effective and that citywide static management was still needed. Universal and enforced control by citywide static management contained 87.65% and 96.29% of transmission opportunities, respectively. The number of daily new and cumulative infections could be significantly reduced if we implemented static management in advance. Moreover, if static management was implemented in the first 14 days of the epidemic, the number of daily new infections would be less than 10. Conclusions: The above research suggests that dynamic zeroing can only be achieved when strict prevention and control measures are implemented as early as possible. In addition, a lot of preparation is still needed if China wants to change its strategy in the future.

## 1. Introduction

Since December 2019, COVID-19 has rapidly spread worldwide [1]. Most countries worldwide implemented comprehensive prevention and control measures in response to the pandemic, some countries followed dynamic zero-COVID strategy to eliminate the virus, while many countries adopted mitigation measures to reduce the number of infections [2,3]. However, SARS-CoV-2 has been evolving in an unanticipated way, which has made prevention and control extremely difficult [4]. Several waves of epidemic resurgences have been caused by the lifting of non-pharmaceutical interventions, the emergence of new variants, and the waning effectiveness of vaccination [5,6,7]. The current dominant variant, i.e., Omicron, was first identified in South Africa in November 2021 [8,9]; it has a shorter incubation period and a more infectious and a stronger immune escape ability [10,11,12,13]. As the global COVID-19 epidemic entered its fourth peak in December 2021, the Omicron variant rapidly replaced the Delta variant as the dominant strain, changing the trajectory of the pandemic [10,14], with China as no exception. Although studies have found a lower proportion of severe illness and deaths after Omicron infection than previous strains, especially in highly vaccinated populations, the high infectivity of Omicron has still led to a large number of hospitalizations, which has increased the pressure on public health, and therefore, efforts are still needed to confront Omicron [15,16,17]. In the face of COVID-19, infectious disease modeling makes an important contribution to public health decision making [18], such as incorporating sociodemographic, vaccination, and other health factors into models to explore the best control strategies for COVID-19 containment [19].

In late February 2022, Shanghai started a new wave of local Omicron epidemic. From that day on, altogether, it took 95 days for Shanghai to lift citywide static management. During this period, the cumulative number of infections was 626,811, which exceeded the total number of previous local Omicron infections in mainland China. Shanghai’s precise control strategy was widely recognized before the Omicron pandemic and had successfully handled its earlier local epidemics. In the beginning, the Shanghai government followed the concept of precise control, but statistics from the Shanghai Municipal Health Commission showed that the trend of the epidemic was beyond people’s imagination. Spreading in the economic and exchange center of China, this wave of epidemic would have a serious impact across the whole country [20]. Just as China responded to the outbreak in Wuhan by adopting lockdown and national emergency response measures to limit the scale of the epidemic [21], the Shanghai government adhered to a dynamic zero-COVID strategy and immediately took a series of more stringent measures to combat the Omicron epidemic. The prevention and control of future epidemics will benefit from the analysis of the Omicron outbreak in Shanghai.

For this round of Omicron epidemic in Shanghai, some studies have been conducted to describe the epidemiological and clinical features of some of the infections [22,23]. Several studies have comprehensively described the epidemiological characteristics, spatiotemporal transmission dynamics, or disease burden of the Omicron outbreak under the population-based screening and lockdown policies implemented in Shanghai [24,25]. There are also some studies that have predicted the Shanghai epidemic situation or have evaluated policy effects through modeling [26,27,28]. However, none of these studies have explored the timing and performance of control measures. If a highly contagious local epidemic caused by imported cases recurs, it is still unclear when and which measures (such as large-scale nucleic acid testing or citywide static management) to take to control the epidemic. To explore the effectiveness of prevention and control measures in different periods and the optimal timing for implementation, we extend a transmission dynamics model based on age structure and vaccination status to fit the epidemic trend in Shanghai and to conduct a counterfactual assessment of possible control strategies and consequences, aiming to provide crucial insights into future epidemic responses. In addition, since the key to prevention and control is infections that are able to move freely in the public space, the extended model takes into consideration both the detected and undetected infection paths, with the undetected proportion reflecting the transmission capacity of the epidemic in different periods.

## 2. Materials and Methods

### 2.1. Data Sources

We collected daily new and cumulative reported cases from the official websites of the Shanghai Municipal Health Commission and the National Health Commission of the People’s Republic of China from 26 February to 31 May 2022, including confirmed cases, asymptomatic infected cases, and asymptomatic cases transferred to confirmed cases. In addition, the age structure of Shanghai’s population in 2020 was obtained from the national census, and the age-specific vaccination coverage in Shanghai was collected up to 10 April 2022 (Table S1).

### 2.2. The Age-Structured and Vaccination-Stratified SEPASHRD Model

**Figure 1:**
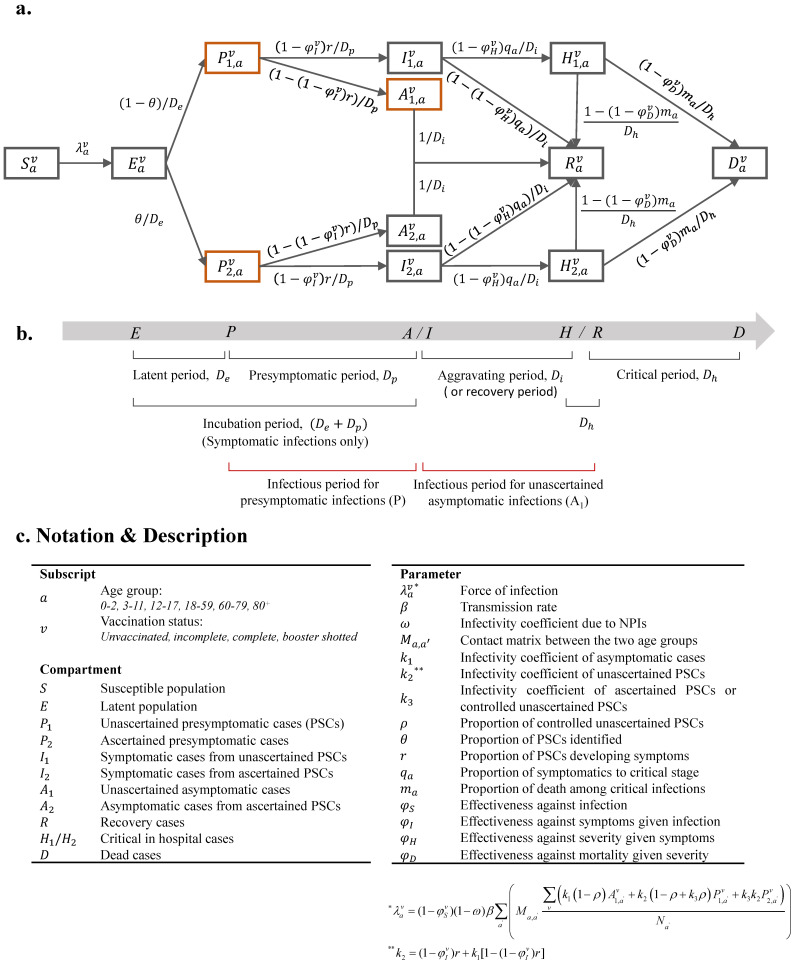
Illustration of the age-structured and vaccination-stratified SEPASHRD model. We extended the classic SEIR model into an age-structured and vaccination-stratified susceptible (S)-exposed (E)-presymptomatic (P)-asymptomatic (A)-symptomatic (S)-hospitalized (H)-recovered (R)-dead (D) model: (**a**) Relationship between different compartments. Four parameters of interest are β (transmission rate), r (asymptomatic rate), θ (ascertainment rate), and ρ (control rate), and the last two parameters are assumed to vary across periods; (**b**) schematic diagram of the course of infection in individuals; (**c**) notation and description of compartments or parameters.

Figure 1 showed the population transmission characteristics of Omicron and the course of the disease in symptomatic individuals. It can be seen that, by extending the SEIR model, we developed a susceptible-exposed-presymptomatic-asymptomatic-symptomatic-hospitalized-recovered-dead (SEPASHRD) model which was stratified by age structure and vaccination status (Figure 1a) to explore the transmission of the Omicron variant in Shanghai. The compartments represent different states of the disease, and three compartments in the model are further divided into two subcompartments: unascertained presymptomatic cases (PSCs, *P*_1_) and ascertained presymptomatic cases (*P*_2_) which are identified by mass nucleic acid screening, unascertained asymptomatic cases from *P*_1_ (*A*_1_) and asymptomatic cases from *P*_2_ (*A*_2_), symptomatic cases from *P*_1_ (*I*_1_) and symptomatic cases from *P*_2_ (*I*_2_). Age structure (0–2, 3–11, 12–17, 18–59, 60–79, and ≥80 years), an age-specific contact matrix for Shanghai during the baseline period [29] (detailed in Table S3 and Figure S1), and vaccination status (unvaccinated, incomplete, complete, and booster shot vaccinated) were considered in the transmission dynamics model. Ordinary differential equations are shown in Table S4. The model structure and parameters are shown in Figure 1b,c.

### 2.3. Parameter Settings and Initial States

The parameter settings for the main analysis are summarized in Tables S5–S9. According to a study by Li et al. [30], we assumed that asymptomatic cases had lower transmissibility and set the relative transmission rate of asymptomatic infections (*k*_1_) as 0.43. The transmissibility of presymptomatic cases were divided into two parts: one part was the same as symptomatic cases and the other part was the same as asymptomatic cases. The proportion of asymptomatic infections of this wave in Shanghai was very high. We assume that the proportion of PSCs developing symptoms (*r*) is 0.093, and the effectiveness against symptomatic infection under different vaccination statuses is $\varphi_{I}^{v}$, therefore, the infectivity coefficient, which represents the transmission ability relative to a symptomatic infection, of PSCs is $k_{2}=(1-\varphi_{I}^{v})r+k_{1}[1-(1-\varphi_{I}^{v})r]$. The infectivity coefficient of unascertained PSCs who have not been controlled in advance is still *k*_2_, but the infectivity coefficient of ascertained PSCs or controlled unascertained PSCs relative to PSCs is *k*_3_, which is fixed as 0 in this study. Therefore, *P*_2_ and part of *P*_1_ are not infectious, the proportion of PSCs identified is *θ* and the proportion of controlled unascertained PSCs is *ρ* (1-*ρ* represents the relative infectivity coefficient of unascertained infections). We assume an incubation period of 3.6 days and a latent period of *D*_e_ = 1.2 days [14,31]. Thus, the presymptomatic infectious period is *D*_p_ = 3.6 − 1.2 = 2.4 days. In addition, the aggravating or recovery period is *D*_i_ = 5.64 days and the critical period is *D*_h_ = 8 days [14]. The disease progression parameters by age groups were taken from the Cai et al. study [14], which were converted to obtain the proportion of symptomatic to the critical stage (*q*_a_) and the proportion of death among critical infections (*m*_a_) (Table S6), and then logit-linear regression fitting was used to obtain the parameters of age groups we needed (Table S7). Vaccine effectiveness assumptions against different clinical outcomes and conditional effectiveness conversion formula were taken from the Cai et al. study [14] (Table S8). The conditional vaccine effectiveness assumptions against different clinical outcomes are shown in Table S9.

According to the seventh national census in China, the total population of Shanghai (*N*) is 24,871,100 [14]. The initial number of susceptible cases *S*(0) can be set according to the age-specific vaccination coverage, as shown in Table S10. In the main analysis, we assume that the initial value of *E* is 10 and randomized to compartments *E* of different ages and vaccination coverages, and the initial values of the other compartments are zero.

### 2.4. Estimation of Parameters in the SEPASHRD Model

Considering the time-varying strength of control measures, we divided the epidemic into four periods according to the implementation of prevention and control measures in Shanghai. The specific division times are as follows: The first period is from 26 February to 15 March 2022; the second period is from 16 March to 3 April 2022; the third period is from 4 April to 21 April 2022; the fourth period is from 22 April to 31 May 2022. We assumed that *θ* and *ρ* were different in the four periods. In our model, the daily reported number of asymptomatic cases was the daily new ascertained PSCs (*P*_2_) and the daily reported number of symptomatic cases was composed of daily new *I*_1_ and *I*_2_. The parameters to be estimated were obtained by fitting the ascertained presymptomatic cases (*P*_2_). Additionally, due to the inaccurate number of symptomatic cases in the early period, only symptomatic cases (*I*) in the fourth period were used for fitting. Except the prior distribution of *β* is lognormal distribution, the other fitting parameters all follow the beta distribution. We explored the parameters to be estimated through Berkeley Madonna and obtained their initial values, which were used as the mean values of the prior distribution. The fourth-order Runge–Kutta method (RK4) was used to solve the differential equations and Markov chain Monte Carlo (MCMC) was used to estimate the parameters.

### 2.5. Simulation Study

To examine potential prevention and control strategies and outcomes from a counterfactual perspective, a number of simulation scenarios were planned, each simulation scenario was built on the premise that one of the periods would be introduced sooner, while others would stay the same, and the simulation would last 150 days. In simulation Scenario 1, the implementation start time of precise control by grid management was advanced. Simulation Scenario 2 advanced the implementation start time of universal control by citywide static management. In simulation Scenario 3, the implementation start time of universal and enforced control by citywide static management was advanced simultaneously. Simulation Scenario 4 advanced the implementation start time of enforced control by citywide static day-by-day management. In addition, the demand and shortage of hospital beds were simulated under different vaccination coverages and control measures.

### 2.6. Statistical Analysis

The Berkeley Madonna Version 10 (Berkeley Madonna Inc., Berkeley, CA, USA) software was used to explore the initial values of the parameters. The R version 4.2.0 (The R Foundation for Statistical Computing, Vienna, Austria) and the R package BayesianTools (version 0.1.8) were used to perform MCMC for 50,000 simulations followed by 20,000 burn-ins. The fitting interval for the number of cases was obtained by adding a random perturbation to the initial value of *E*. Unless specified otherwise, all parenthetical ranges refer to the 95% credible interval.

## 3. Results

### 3.1. Brief Description of the Shanghai Omicron Epidemic

**Figure 2:**
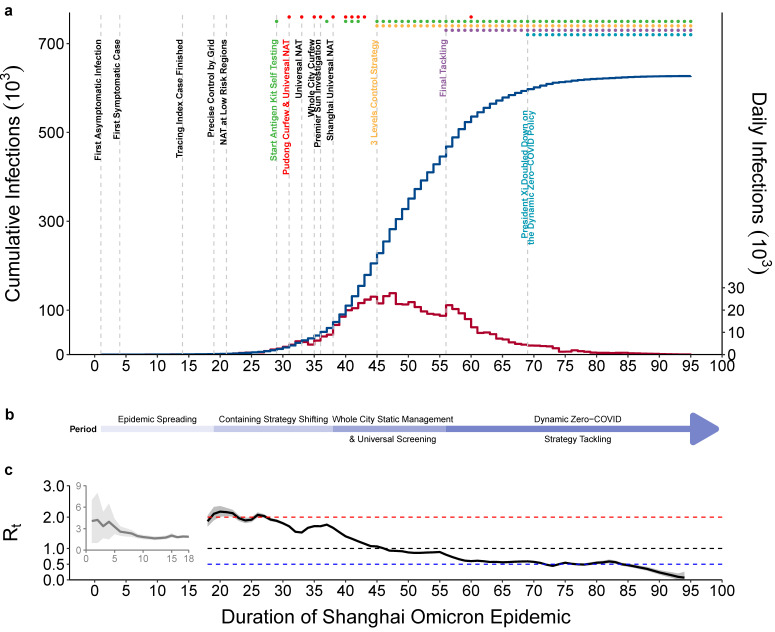
Progress of the Shanghai omicron epidemic and major events between 26 February and 31 May 2022: (**a**) Progress and major events of the Omicron outbreak in Shanghai. The blue and red stairs represent cumulative and daily new positive cases, respectively. The top of the picture marks the major events or implementation measures of the epidemic in Shanghai. The red dot represents the universal nucleic acid test, the green dot represents antigen detection, the orange dot represents the 3-level control strategy, the purple dot represents the final attack, and the blue dot represents doubling down on the dynamic Zero-COVID policy; (**b**) four periods of the epidemic in Shanghai according to the prevention and control policies; (**c**) estimated *R*_t_ between 26 February and 31 May 2022.

Under the influence of a surge in imported infections, the Omicron variant has been cryptically spreading in Shanghai. The Shanghai government implemented a series of prevention and control measures to contain the outbreak after the first reported positive case was found. Figure 2a showed the progress of the Shanghai Omicron epidemic and the important events and major control measures implemented by the government between 26 February and 31 May 2022. Based on the development of the epidemic and the implementation time of some control measures, the epidemic was divided into four periods: epidemic spreading, containing strategy shifting, whole city static management and universal screening, and dynamic zero-COVID strategy tackling (Figure 2b). The estimated time-varying regeneration number, which told us the average number of people who will catch a disease from one contagious person at time *t*, was plotted to reflect the change of transmission capacity. The first period was the spread of the epidemic from 26 February to 15 March 2022, during which *R*_t_ was high at the beginning, and then rapidly increased after rapid suppression. During the second period from 16 March to the solstice on 3 April 2022, the control policy was changed to contain the outbreak. On March 16, precise control by grid management was implemented as the starting point of this period, followed by a series of large-scale nucleic acid tests and regional static management. However, the number of positive cases was still rising rapidly, and *R*_t_ fluctuated around 2. During the third period, from 4 April to 21 April 2022, citywide static management and universal nucleic acid screening were carried out in an organized manner. In this stage, the daily number of new positive cases was relatively stable and *R*_t_ floated around one. During the fourth period from 22 April to 31 May 2022, the city entered the community zero elimination stage. All measures were implemented more strictly, and the number of cases was in a period of rapid decline, *R*_t_ < 1 (Figure 2).

### 3.2. Model Design, Fitting, and Parameter Estimations

**Figure 3:**
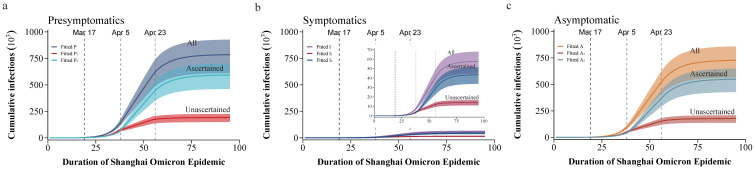
Modeling the Omicron epidemic in Shanghai. The Shanghai Omicron epidemic trend fitted by the SEPASHRD dynamics model: (**a**) The cumulative number of presymptomatic infections fitted by the model; (**b**) the cumulative number of symptomatic infections fitted by the model; (**c**) the cumulative number of asymptomatic infections fitted by the model. The shaded areas in (**a**–**c**) are 95% credible intervals of 2000 simulations, and the colored solid lines are the median values based on 30,000 MCMC samples.

The fitted daily new ascertained presymptomatic cases over the whole course and symptomatic cases in the fourth period agreed well with the observed data (Figure S2). The reported daily new infections during the first three periods were lower than model predictions, which resulted in the fitted cumulative ascertained presymptomatic cases and symptomatic infections being higher than the reported numbers from the government. Figure 3 shows the presymptomatic, symptomatic, and asymptomatic cumulative infections obtained through model fitting. As of 31 May 2022, the fitted cumulative number of infections was 785,123 (95% CI 611,105–927,282), of which 592,921 (95% CI 461,751–701,591) was ascertained (Figure 3a). The fitted cumulative number of symptomatic infections was 57,585 (95% CI 448,428–679,855), of which 24.51% were unascertained in the presymptomatic phase (Figure 3b). The fitted cumulative number of asymptomatic infections was 726,834 (95% CI 565,741–858,444), of which 24.49% were undetected (Figure 3c). In addition, according to the daily new infections, the number during the first two periods of the epidemic was in rapid increase, while the third period was a sustaining period. In the final period, the number of daily new infections began to decline rapidly, and the epidemic was under control (Figure S2a–c).

The parameters fitted by MCMC converged, indicating that the results are reliable. The detailed parameter values are shown in Figure S3. We estimated the transmission rate (*β*) to be 0.3809 (0.3758–0.3867). The proportion of ascertained PSCs (*θ*) in the four periods gradually increased, which partly reflected the intensity of implementing large-scale nucleic acid testing and other measures, and was 38.92% (38.01–39.91%), 45.42% (45.13–45.73%), 78.07% (77.95–78.20%), and 89.98% (89.60–92.34%), respectively. This meant that, in the early period of the epidemic, about 40% of the infections had been detected in the presymptomatic stage as compared with more than 75% in the later period. Some of the unascertained PSCs were controlled in advance (*ρ*), fixed to 0 in the first period, and the estimated values of the remaining three periods were 41.66% (40.88–42.37%), 43.69% (42.79–44.56%), and 63.01% (62.26–63.87%), respectively. This meant that even if some of the infections were not detected in the presymptomatic stage, more than 40% of them had been controlled and were not contagious.

### 3.3. The Real Situation of the Shanghai Omicron Epidemic

**Figure 4:**
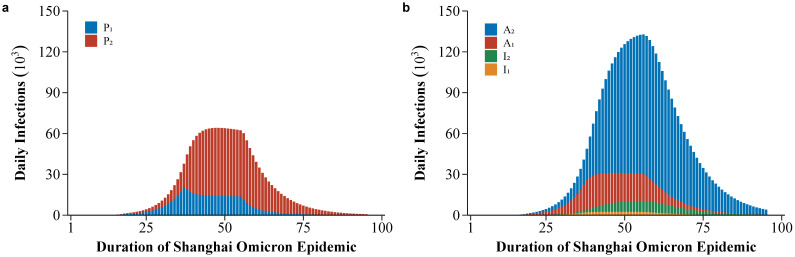
Estimated number of active infectious cases in Shanghai from 26 February to 31 May 2022: (**a**) Estimated number of active presymptomatic infections. The blue bars represent the unascertained presymptomatic cases (PSCs) (the red bars represent the ascertained presymptomatic cases); (**b**) estimated number of active symptomatic or asymptomatic infections. The red bars represent the unascertained asymptomatic cases and the red bars represent the asymptomatic cases from ascertained PSCs. The orange bars represent symptomatic cases from unascertained PSCs and the green bars represent symptomatic cases from ascertained PSCs.

The model also projected the number of daily active infections. The specific number of daily active presymptomatic, symptomatic, and asymptomatic infections are shown in Figure 4. The number of daily active presymptomatic infections (*P*_1_ and *P*_2_) peaked at 64,102 on 14 April 2022, and then declined to 704 on 31 May 2022 (Figure 4a). The number of daily active *I*_1_ peaked at 2465 on 13 April 2022 and decreased to 35 on 31 May 2022. Similarly, the number of daily active *I*_2_ peaked at 8148 on 23 April 2022 and decreased to 291 on 31 May 2022. As can be seen from the figure, the proportion of ascertained infections was higher than that of unascertained infections, and the proportion of asymptomatic infections was higher than that of symptomatic infections.

### 3.4. Counterfactual Evaluation of Possible Control Strategies and Consequences

**Figure 5:**
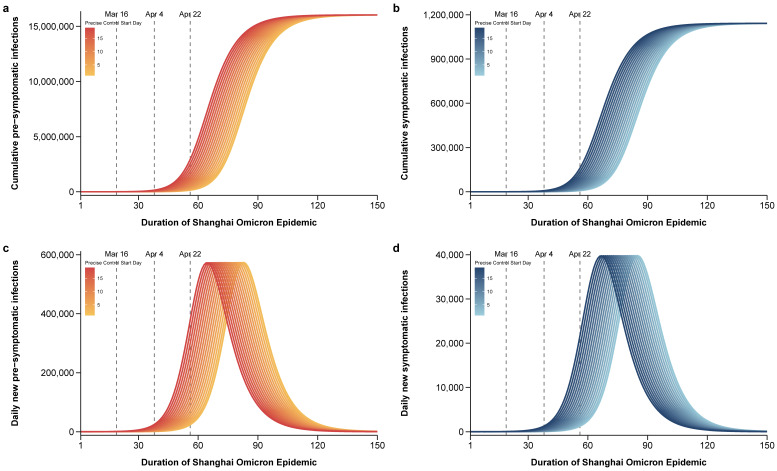
Outcomes of starting precise control by grid management at different time points and using it continuously. The final scale of the epidemic situation within 150 days in which precise control by grid management was implemented from the 1st day to the 19th day of the epidemic: (**a**) Cumulative number of positive infections; (**b**) cumulative number of symptomatic infections; (**c**) daily new number of positive infections; (**d**) daily new number of symptomatic infections.

The Shanghai government adopted a series of targeted intervention and management strategies, known as precise control by grid management, from 16 March 2022 to the full lockdown of the city (the second period). Figure 5 simulates the final scale of the epidemic at different time points after continuously implementing precise control by grid management. If precise control by grid management was adopted from March 16 to the end, the epidemic would not have been under control, and the cumulative number of presymptomatic infections would have exceeded 16 million (Figure 5a) and the cumulative number of symptomatic infections would have exceeded 1 million after 150 days of epidemic progression (Figure 5b). Even if the start time of the precise control by grid implementation was advanced day-by-day, the growth momentum would not be suppressed, and the final cumulative number of infections would almost be the same, but would slightly delay the peak time (Figure 5).

**Figure 6:**
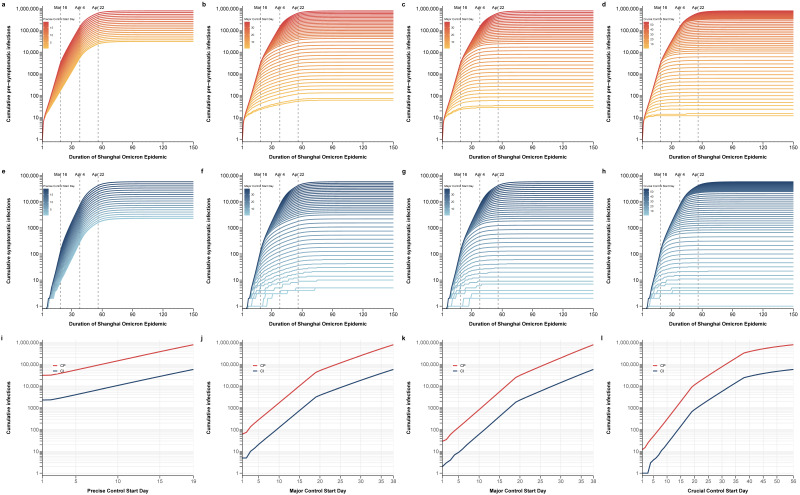
The scale of the epidemic after different controls were implemented at different times. All the results were obtained under the premise that the implementation time of prevention and control measures at one period was advanced day by day, while the other periods remained unchanged: (**a**,**e**,**i**) Advancing precise control by grid management day by day; (**b**,**f**,**j**) advancing the implementation start time of universal control by citywide static management; (**c**,**g**,**k**) advancing the implementation start time of universal and enforced control by citywide static management simultaneously; (**d**,**h**,**l**) advancing the implementation start time of enforced control by citywide static management. (**a**–**d**) cumulative number of positive infections, (**e**–**h**) cumulative number of symptomatic infections, (**i**–**l**) the final scale of the outbreak after 150 days under different control measures.

**Figure 7:**
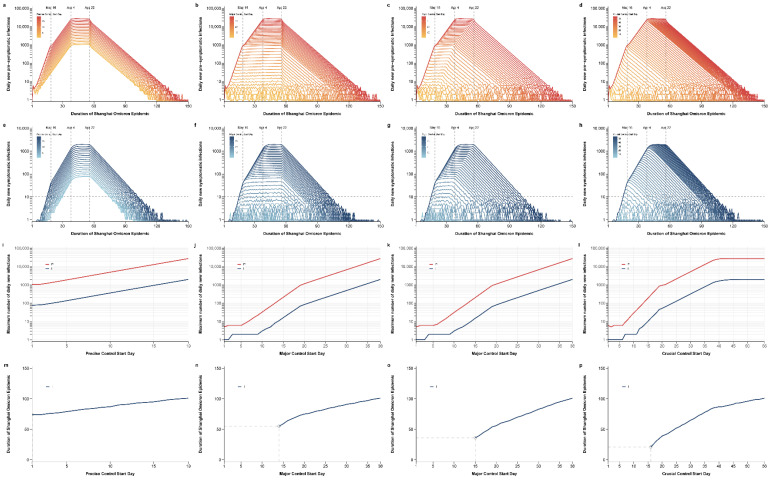
Duration of the epidemic after different controls were implemented at different times. All the results were obtained under the premise that the implementation time of prevention and control measures at one period was advanced day by day, while the other periods remained unchanged: (**a**,**e**,**i**,**m**) Advancing precise control by grid management day by day; (**b**,**f**,**j**,**m**) advancing the implementation start time of universal control by citywide static management; (**c**,**g**,**k**,**o**) advancing the implementation start time of universal and enforced control by citywide static management simultaneously; (**d**,**h**,**l**,**p**) advancing the implementation start time of enforced control by citywide static management. (**a**–**d**) Daily new number of positive infections, (**e**–**h**) daily new number of symptomatic infections, (**i**–**l**) the maximum number of daily new infections within 150 days under different control measures, (**m**–**p**) duration of the epidemic under different prevention and control strategies within 150 days. (Fewer than 10 daily new symptomatic infections are considered to be the end of the epidemic.)

Next, we conducted a counterfactual assessment of possible control strategies and consequences to explore three potential control modes. Simulating the scale and duration of the epidemic after the implementation of a certain period’s strategy was advanced while maintaining the original control strategy in other periods. The final epidemic scale under different strategies is shown in Figure 6, and the duration of the epidemic is detailed in Figure 7 (the simulation duration was 150 days).

Simulation Scenario 1 only advances day-by-day precise control by grid management. The early implementation of second period measures has a slight impact on the final cumulative number of infections (Figure 6a,e), the peak of daily new infections (Figure 7a,e), and the duration of the epidemic (the standard for the end of an epidemic is less than 10 new symptomatic infections per day) (Figure 7m), but it could still result in a large-scale epidemic. Even if grid precision control was implemented from the first day, the cumulative number of presymptomatic and symptomatic infections could reach up to 30,991 and 2280, respectively (Figure 6i), and the peak of daily new presymptomatic and symptomatic infections would be 1053 and 77 (Figure 7i).

Simulation Scenarios 2 and 3, respectively, advance the implementation start time of universal control by citywide static management and the implementation start time of universal and enforced control by citywide static management advanced simultaneously. The cumulative number of infections and the peak of daily new infections gradually decreased with the advance of the citywide static measures (Figure 6b,c,f,g). If universal control under citywide static management is implemented in advance to the first day, the cumulative number of presymptomatic infections is 62 and the cumulative number of symptomatic infections is 5 (Figure 6j,k). The universal control by citywide static management can quickly suppress the peak, and the peak of daily new infections gradually decreases with the advance of these measures (Figure 7b,c,f,g,j,k). Enforced control by citywide static management can quickly contain the epidemic and shorten the duration of the epidemic, and therefore, the duration of the epidemic in Scenario 3 is shortened faster than that in Scenario 2 (Figure 7n,o). If universal control by citywide static management is implemented before the 14th day of the outbreak, it would prevent a pandemic (Figure 7n,o).

Simulation Scenario 4 advances the implementation start time of day-by-day enforced control by citywide static management. The cumulative number of infections declines rapidly as implementation started earlier (Figure 6b,h,l). The peak of daily new infections remains unchanged when the implementation of enforced control is later than that of universal control by citywide static management, but declines rapidly once it is earlier (Figure 7d,h,l). Under this scenario, the duration of the epidemic also decreases rapidly, and the implementation of this measure before the 16th day of the epidemic would prevent a large-scale epidemic (Figure 7p).

Finally, with only basic public health prevention and control measures, or with the first period of control measures, almost everyone becomes infected. With the existing vaccination coverage in Shanghai (Table S1) and basic public health prevention and control measures (without additional control measures in our model), there would be 17 days of hospital beds occupancy exceeding the threshold of 25,000 [32], and the maximum number of beds required for COVID-19 infection would be 40,036. Even if the first period control measures were maintained, there would not be enough to avoid resource collapse. However, if vaccination coverage was increased to the level in Hong Kong as of 24 September 2022 [33], especially among the elderly (Table S2), overloading hospital beds occupancy could be avoided. (Figure S4).

## 4. Discussion

This study used an extended dynamics model to comprehensively explore the transmission of Omicron in Shanghai in 2022. The relevant parameters involved in this model reflected the strength of prevention and control measures at different periods of this epidemic in Shanghai. It is clear that the focused and precise interventions implemented during the early stages of the outbreak were insufficient to stop the virus from spreading, and the pandemic could only be successfully contained by implementing some strong actions after the entire city was placed under static management. Our study shows that precise control by grid management can only contain 68.16% of transmission opportunities, while universal control by citywide static management can contain 87.65% of transmission opportunities, and enforced control by citywide static management can contain more than 95% of transmission opportunities.

The rapid outbreak in Shanghai highlights several key features of Omicron, i.e., high transmissibility and/or immune escape and high asymptomatic carriage [34,35,36]. According to the actual data of Shanghai, 94.3% of all new cases were asymptomatic on first diagnosis [37]. Our model assumed that the symptomatic rate was *r* = 9.3%, that is, the estimated asymptomatic proportion was 90.7% [25]. This high asymptomatic proportion is related to the characteristics of the Omicron variant. Additionally, the partial effect of COVID-19 vaccination has resulted in some people not developing symptoms even after being infected [12], which makes the spread of Omicron epidemic more insidious. The high covertness emphasizes the importance of early detection, in which nucleic acid testing is a vital measure to quickly detect infected people including those with no symptoms, timely locking the scope and target of control, and then taking measures such as isolation to cut off the transmission route [38,39]. It is also a key measure to realize the “four early” measures, namely early detection, early reporting, early isolation, and early treatment [40]. Moreover, reviewing China’s response to local outbreaks shows that mass, community-wide nucleic acid testing has proven to be effective [39].

Our findings highlight the failure of the precise containment measures implemented in Shanghai in March to stop the spread of the outbreak. If appropriate measures are not implemented within an optimal intervention time, the window period will be missed, leading to high caseloads as well as resource crowding in testing and contact tracing capacity, and further leading to a decline in the effectiveness of targeted interventions and faster epidemic growth [41]. If strong measures are implemented in a short period, they could prevent a pandemic. Studies have proposed how containment could be achieved and maintained in China, mainly including accelerating case identification and preventing onward transmission [41,42]. A major strength of this paper is that the model takes into consideration the proportion of presymptomatic cases identified at each period and the proportion of unascertained presymptomatic cases under control, which can reflect the effectiveness of prevention and control at each stage of the epidemic in Shanghai from the side. The proportion of unascertained cases plays a crucial role in prioritizing surveillance or other control measures [43]. Symptom-based surveillance in medical institutions and communities, routine screening of key populations, and border control policies are routine interventions before outbreaks [42,44]. Contact tracing and large-scale nucleic acid testing are relevant measures to accelerate the identification of cases after the outbreak [39,45]. In our model, the detection ability is mainly reflected in the parameter *θ*, and it can be seen that the proportion of cases identified in the four periods has gradually increased. Measures such as isolation of close contacts, residential community confinement, and mobility restrictions can interrupt the chain of transmission [41,42], and their effectiveness can be partly reflected in the parameter *ρ*. With the implementation of static management measures in the whole city, people’s movements are restricted, and the proportion of quarantine increases.

Although the regional static management implemented in Shanghai prevented further development of the epidemic and successfully achieved containment, the huge cost of such a prolonged lockdown was extremely detrimental to the operation and development of the city [24], therefore, it is particularly important to grasp the time window for intervention. This study’s simulation of the intervention time window and counterfactual analysis of potential prevention and control strategies and their effects are further highlights. From our simulation results, it can be seen that the implementation of high-intensity isolation measures and mass nucleic acid testing within 2 weeks after the detection of an epidemic could prevent an outbreak. The earlier the interventions are implemented, the shorter the duration of the outbreak. We found that Shanghai’s public budget revenue fell 8.9% year-on-year in January–July due to the pandemic, while expenditure rose 4.8% year-on-year [46]. Zhang et al. [47] found that social distancing incurred a daily cost of USD 377 million, in 2020, in Shanghai. Therefore, this study also discussed the burden of hospitalization if the policy is changed, which is another strength of this paper.

We acknowledge that this study has some limitations. First of all, we only used the publicly reported cumulative number of symptomatic and asymptomatic infections, and the data may be inaccurate, especially the first three periods of reported symptomatic infections were significantly lower than the model predictions, which was affected by delayed reporting and insufficient detection capacity. The incompleteness of the data prevented us from evaluating the burden of disease, including the number of severe cases and deaths, etc. Second, in addition to considering age-structured contact patterns and different vaccine protection efficacy, our model ignores the heterogeneity between groups or individuals with different gender, geographic region, and economic statuses [48,49], and only takes into consideration homogenous transmission among populations. Furthermore, this model does not consider the impact of increasing the proportion of vaccination on changing the time window of intervention or duration and magnitude of the epidemic. Studies have shown that although vaccine effectiveness against infection is weak, it provides high effectiveness against severe consequences [34,50,51]. For example, a study of real-world data in Hong Kong showed that the effectiveness against mild or moderate disease in adults aged 20–59 years was only estimated to be 25.1%, while the effectiveness against severe disease and death was over 90%. Although the effectiveness in the elderly group had decreased, it was also over 60%, and the booster was even more protective [52]. According to the data from Shanghai, the coverage of complete vaccination was as high as 86.52%, but only 13.76% of people over 80 years old had received two doses of the vaccine. Moreover, our study showed that under the vaccination coverage in Shanghai on 10 April 2022, without strict intervention measures, there would have been a severe strain on bed resources. Therefore, while establishing a perfect emergency response system, it is still necessary to promote administering vaccines, especially for vulnerable groups such as the elderly. In the future, accelerating the vaccination coverage of the elderly population and improving the stock of medical resources, especially ICU beds and equipment, will be the key to shifting from local containment to long-term mitigation.

## 5. Conclusions

In conclusion, in the face of the Omicron epidemic, the effectiveness of Shanghai’s precise prevention and control mode is limited. Dynamic zeroing can be achieved only by implementing a series of strong prevention and control measures as soon as possible. In addition, in the future, the strategic focus could shift from prevention of infection to prevention of severe or critical illness. If China is going to move away from local containment and into long-term mitigation, the results of this study can give an indication of the efforts needed.

## Data Availability

The relevant data of the model are provided in the supplementary information. Publicly available datasets were analyzed in this study. This data can be found here: https://wsjkw.sh.gov.cn/yqtb/index.html (accessed 31 on December 2022).

## Supplementary Materials

The following supporting information can be downloaded at: https://www.mdpi.com/article/10.3390/tropicalmed8010039/s1, Figure S1. Age-mixing patterns in Shanghai during baseline period; Figure S2. Trend of model fitted and observed daily new infections in Shanghai Omicron epidemic; Figure S3. Trace plot of estimated parameters from the main analyses by MCMC and violin diagram of parameters; Figure S4. Projected demand and shortage of hospital beds under four different scenarios; Table S1. Age structure of Shanghai’s population in 2020 was obtained from the Chinese Census and age-specific vaccination coverage in Shanghai up to the outbreak of Omicron epidemic (as of 10 April 2022); Table S2. Age-specific vaccination coverage in simulation study was consistent with the situation in Hong Kong on 24 September; Table S3. Mean normalized contact matrix in Shanghai during baseline period; Table S4. Transmission Dynamics Model Differential Equations for Shanghai Omicron Epidemic Replay; Table S5. Parameter settings for four periods in the main analysis; Table S6. Parameters of disease progression by age group from Cai J publication; Table S7. Parameters of disease progression by age group in main analysis; Table S8. Vaccine effectiveness assumptions against different clinical outcomes and conditional effectiveness conversion formula from Cai J publication; Table S9. Conditional vaccine effectiveness assumptions against different clinical outcomes; Table S10. Initial values of dynamic model compartments. Reference [53] is cited in the supplementary materials.
