# Supplementary material for: Retrospective Modeling of the Omicron Epidemic in Shanghai, China: Exploring the Timing and Performance of Control Measures
Source: Trop Med Infect Dis. 2023 Jan 5;8(1):39. doi: 10.3390/tropicalmed8010039 (PMC9862922; doi:10.3390/tropicalmed8010039)
Supplement: Supplementary file 1 [file tropicalmed-08-00039-s001.zip › tropicalmed-2110839-supplementary.pdf]

Supplementary Materials

Retrospective Modeling of the Omicron Epidemic in Shanghai, China: Exploring the Timing and Performance of Control Measures

Lishu Lou [1,†], Longyao Zhang [1,†], Jinxing Guan [1], Xiao Ning [1], Mengli Nie [1], Yongyue Wei [1,2,*] and Feng Chen [1,*]

1 Department of Biostatistics, School of Public Health, Center of Global Health, Nanjing Medical University, Nanjing 211166, China
2 Center for Public Health and Epidemic Preparedness & Response, Peking University, Xueyuan Road, Haidian District, Beijing 100191, China
* Correspondence: ywei@bjmu.edu.cn (Y.W.); fengchen@njmu.edu.cn (F.C.); Tel.: +86-025-86868414 (F.C.)
† These authors contributed equally to this work.

Table of Contents

Supplementary Tables 3

Table S1. Age structure of Shanghai's population in 2020 was obtained from the Chinese Census and age-specific vaccination coverage in Shanghai up to the outbreak of Omicron epidemic (as of April 10, 2022). 3

Table S2. Age-specific vaccination coverage in simulation study was consistent with the situation in Hong Kong on September 24. 3

Table S3. Mean normalized contact matrix in Shanghai during baseline period. 3

Table S4. Transmission Dynamics Model Differential Equations for Shanghai Omicron Epidemic Replay. 4

Table S5. Parameter settings for four periods in the main analysis. 5

Table S6. Parameters of disease progression by age group from Cai J publication. 5

Table S7. Parameters of disease progression by age group in main analysis. 5

Table S8. Vaccine effectiveness assumptions against different clinical outcomes and conditional effectiveness conversion formula from Cai J publication. 6

Table S9. Conditional vaccine effectiveness assumptions against different clinical outcomes. 6

Table S10. Initial values of dynamic model compartments. 7

Supplementary Figures 8

Figure S1. Age-mixing patterns in Shanghai during baseline period. 8

Figure S2. Trend of model fitted and observed daily new infections in Shanghai Omicron epidemic. 9

Figure S3. Trace plot of estimated parameters from the main analyses by MCMC and violin diagram of parameters. 10

Figure S4. Projected demand and shortage of hospital beds under four different scenarios. 11

Reference 12

Supplementary Tables

Table S1. Age structure of Shanghai's population in 2020 was obtained from the Chinese Census and age-specific vaccination coverage in Shanghai up to the outbreak of Omicron epidemic (as of April 10, 2022)

Age group	Population	No vaccination coverage (%)	Incomplete vaccination coverage (%)	Complete vaccination coverage (%)	Booster vaccination coverage (%)
0-2	454600	100.00	0.00	0.00	0.00
3-11	1610700	31.56	8.56	59.88	0.00
12-17	714700	2.55	2.18	95.27	0.00
18-59	16275500	0.00	0.00	100.00	57.52
60-79	4979700	28.48	1.66	69.87	42.51
80+	835900	85.54	0.70	13.76	7.97
Overall	24871100	12.52	0.97	86.51	46.42

Table S2. Age-specific vaccination coverage in simulation study was consistent with the situation in Hong Kong on September 24 [1]

Age group	No vaccination coverage (%)	Incomplete vaccination coverage (%)	Complete vaccination coverage (%)	Booster vaccination coverage (%)
0-2	83.56	16.44	0.00	0.00
3-11	15.71	14.09	70.20	20.96
12-17	0.00	2.04	97.96	61.31
18-59	0.00	0.00	100.00	81.28
60-79	13.04	0.99	85.97	77.28
80+	28.93	2.84	68.23	55.07
Overall	6.13	1.56	92.31	73.63

Table S3. Mean normalized contact matrix in Shanghai during baseline period

<table>
<tr><td colspan="2" rowspan="2"></td><td colspan="6">Age of contact</td></tr>
<tr><td>0-2</td><td>3-11</td><td>12-17</td><td>18-59</td><td>60-79</td><td>80+</td></tr>
<tr><td rowspan="6">Age of participant</td><td>0-2</td><td>0.81</td><td>0.37</td><td>0.00</td><td>0.21</td><td>0.09</td><td>0.16</td></tr>
<tr><td>3-11</td><td>0.37</td><td>2.71</td><td>1.59</td><td>0.27</td><td>0.14</td><td>0.24</td></tr>
<tr><td>12-17</td><td>0.00</td><td>1.59</td><td>4.97</td><td>0.64</td><td>0.12</td><td>0.34</td></tr>
<tr><td>18-59</td><td>0.21</td><td>0.27</td><td>0.64</td><td>1.22</td><td>0.35</td><td>0.64</td></tr>
<tr><td>60-79</td><td>0.09</td><td>0.14</td><td>0.12</td><td>0.35</td><td>1.12</td><td>2.63</td></tr>
<tr><td>80+</td><td>0.16</td><td>0.24</td><td>0.34</td><td>0.64</td><td>2.63</td><td>4.99</td></tr>
</table>

The source data are from Zhang J [2] publication. The original matrix is annualized and then compressed into the mean normalized matrix with specified age groups following the method introduced in the previous publication [3].

Table S4. Transmission Dynamics Model Differential Equations for Shanghai Omicron Epidemic Replay

Model	Formula
Transmission dynamics model	$$\begin{cases} \frac{dS_a^v}{dt} = -\lambda_a^v S_a^v \\ \frac{dE_a^v}{dt} = \lambda_a^v S_a^v - \frac{1}{D_e} E_a^v \\ \frac{dP_{1,a}^v}{dt} = \frac{(1-\theta)}{D_e} E_a^v - \frac{1}{D_p} P_{1,a}^v \\ \frac{dI_{1,a}^v}{dt} = \frac{(1-\varphi_I^v) r}{D_p} P_{1,a}^v - \frac{1}{D_i} I_{1,a}^v \\ \frac{dH_{1,a}^v}{dt} = \frac{(1-\varphi_H^v) q_a}{D_i} I_{1,a}^v - \frac{1}{D_h} H_{1,a}^v \\ \frac{dA_{1,a}^v}{dt} = \frac{1-(1-\varphi_I^v) r}{D_p} P_{1,a}^v - \frac{1}{D_i} A_{1,a}^v \\ \frac{dR_a^v}{dt} = \frac{1-(1-\varphi_H^v) q_a}{D_i} (I_{1,a}^v + I_{2,a}^v) + \frac{1-(1-\varphi_D^v) m_a}{D_h} (H_{1,a}^v + H_{2,a}^v) + \frac{1}{D_i} (A_{1,a}^v + A_{2,a}^v) \\ \frac{dD_a^v}{dt} = \frac{(1-\varphi_D^v) m_a}{D_h} (H_{1,a}^v + H_{2,a}^v) \\ \frac{dP_{2,a}^v}{dt} = \frac{\theta}{D_e} E_a^v - \frac{1}{D_p} P_{2,a}^v \\ \frac{dA_{2,a}^v}{dt} = \frac{1-(1-\varphi_I^v) r}{D_p} P_{2,a}^v - \frac{1}{D_i} A_{2,a}^v \\ \frac{dI_{2,a}^v}{dt} = \frac{(1-\varphi_I^v) r}{D_p} P_{2,a}^v - \frac{1}{D_i} I_{2,a}^v \\ \frac{dH_{2,a}^v}{dt} = \frac{(1-\varphi_H^v) q_a}{D_i} I_{2,a}^v - \frac{1}{D_h} H_{2,a}^v \\ N = \sum_{a,v} S_a^v + E_a^v + P_{1,a}^v + I_{1,a}^v + A_{1,a}^v + H_{1,a}^v + P_{2,a}^v + I_{2,a}^v + A_{2,a}^v + H_{2,a}^v + R_a^v + D_a^v \end{cases}$$
Force of infection	$$\lambda_a^v = (1-\varphi_S^v)(1-\omega)\beta \sum_{a'} \left(M_{a,a'} \frac{\sum_v \left(k_1 (1-\rho) A_{1,a'}^v + k_2 (1-\rho + k_3 \rho) P_{1,a'}^v + k_3 k_2 P_{2,a'}^v \right)}{N_{a'}} \right)$$

$$k_2 = (1-\varphi_I^v) r + k_1 [1 - (1-\varphi_I^v) r]$$

Table S5. Parameter settings for four periods in the main analysis

Parameter	Day 1-18 (Feb 26-Mar 15)	Day 19-37 (Mar 16-Apr 3)	Day 38-55 (Apr 4-Apr 21)	Day 55-95 (Apr 22-May 31)	Range
β	β	β	β	β	Fitted
ω	ω_1	ω_2	ω_3	ω_4	Fixed as 0 for real data fitting
$M_{a,a'}$	$M_{a,a'}$	$M_{a,a'}$	$M_{a,a'}$	$M_{a,a'}$	Fixed [2]
k_1	0.43	0.43	0.43	0.43	Fixed [4]
k_2*	k_2	k_2	k_2	k_2	Fitted
k_3	k_3	k_3	k_3	k_3	Fixed as 0 for real data fitting
ρ	ρ_1=0	ρ_2	ρ_3	ρ_4	Fitted
θ	θ_1	θ_2	θ_3	θ_4	Fitted
r	0.093	0.093	0.093	0.093	Fixed [5]
D_e	1.2	1.2	1.2	1.2	Fixed [6]
D_p	2.4	2.4	2.4	2.4	Fixed [7]
D_i	5.64	5.64	5.64	5.64	Fixed [6]
D_h	8	8	8	8	Fixed [6]
N	24871100	24871100	24871100	24871100	Fixed

a' represents the age group of the people who comes into contact with age group a

$$k_2{*} = (1-\varphi_I^v)r + k_1[1-(1-\varphi_I^v)r]$$

Table S6. Parameters of disease progression by age group from Cai J [6] publication

Parameter	Age group								
	0-2	3-11	12-17	18-29	30-39	40-49	50-59	60-69	70+
p_a^h*	11.09	6.37	15.01	7.41	9.78	8.02	17.06	20.45	65.60
p_a^{icu}	0.23	1.61	3.33	5.39	9.82	14.66	19.49	29.67	48.22
p_a^{HD}	0.26	0.52	0.75	0.96	0.80	3.23	5.75	8.68	24.98
p_a^{UD}	3.40	6.76	9.81	12.48	1045	20.66	33.32	36.45	37.47
m_a**	0.27	0.62	1.05	1.58	1.75	5.79	11.12	16.92	31.00

* $q_a = p_a^h$ ** $m_a = p_a^{icu} * p_a^{UD} + (1 - p_a^{icu}) * p_a^{HD}$

Table S7. Parameters of disease progression by age group in main analysis

Parameter	Age group					
	0-2	3-11	12-17	18-59*	60-79*	80+*
q_a	11.09	6.37	15.01	15.18	29.70	42.71
m_a	0.27	0.62	1.05	3.50	19.10	44.92

* Logit-linear regression fitted using data in Supplementary Table 6 is used to predict the q- and m-values for the age group 18-59, 60-79, and 80+.

Table S8. Vaccine effectiveness assumptions against different clinical outcomes and conditional effectiveness conversion formula from Cai J [6] publication

Effectiveness against	Inactivated incomplete vaccination	Inactivated complete vaccination	Inactivated booster vaccination
Infection(ϵ_{inf})	5.6%	9.1%	17.0%
Symptom(ϵ_{symp})	16.5%	26.9%	46.5%
Severity(ϵ_{sever})	46.2%	78.8%	98.1%
Mortality(ϵ_{mortal})	56.3%	83.2%	98.4%

Table S9. Conditional vaccine effectiveness assumptions against different clinical outcomes

Notation	Conditional effectiveness against	Calculation*	Inactivated incomplete vaccination	Inactivated complete vaccination	Inactivated booster vaccination
φ_S^v	Infection		5.6%	9.1%	17%
φ_I^v	symptoms given infection	$\epsilon_{symp\|inf} = \frac{\epsilon_{symp} - \epsilon_{inf}}{1 - \epsilon_{inf}}$	11.55%	19.58%	35.54%
φ_H^v	severity given symptoms	$\epsilon_{sever\|symp} = \frac{\epsilon_{sever} - \epsilon_{symp}}{1 - \epsilon_{symp}}$	35.57%	71.00%	96.45%
φ_D^v	mortality given severity	$\epsilon_{mortal\|sever} = \frac{\epsilon_{mortal} - \epsilon_{sever}}{1 - \epsilon_{sever}}$	18.77%	20.75%	15.79%

* The above vaccine effectiveness are calculated according to Supplementary Table 8

Table S10. Initial values of dynamic model compartments

Compartment	Vaccination status	Initial value*					
		Age group					
		0-2	3-11	12-17	18-59	60-79	80+
S	No vaccination	454600	508324	18194	0	1417978	715029
	Incomplete vaccination	0	137826	15596	0	82474	5845
	Complete vaccination	0	964550	680910	6913785	1362224	48390
	Booster vaccination	0	0	0	9361715	2117024	66636
E		10					
N overall		24871100					

* Initial values of all the compartments not listed in the table were zero.

Supplementary Figures

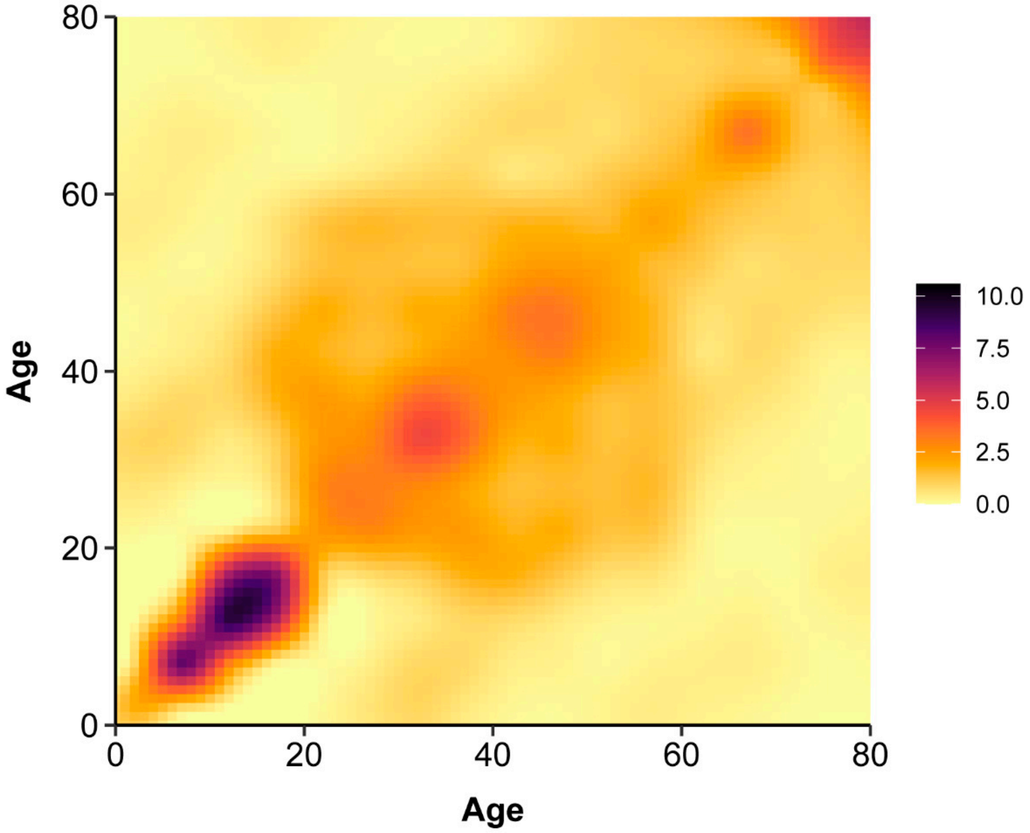

Figure S1. Age-mixing patterns in Shanghai during baseline period

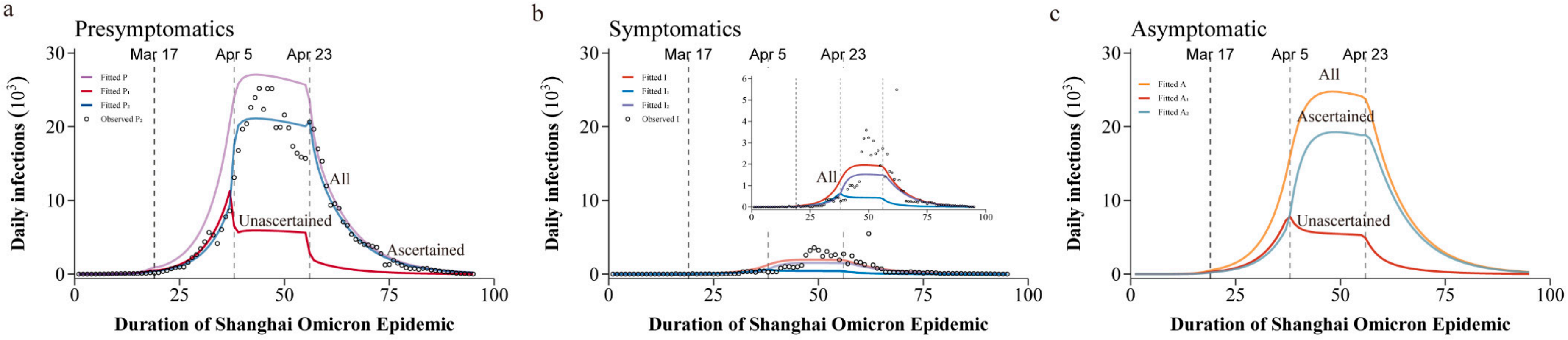

Figure S2. Trend of model fitted and observed daily new infections in Shanghai Omicron epidemic. Shanghai Omicron epidemic trend fitted by the SEPASHRD dynamics model. a. The daily new pre-symptomatic infections fitted by the model. b. The daily new symptomatic infections fitted by the model. c. The daily new asymptomatic infections fitted by the model. Circles represent actual values, and the coloured solid lines are the median values based on 30,000 MCMC samples.

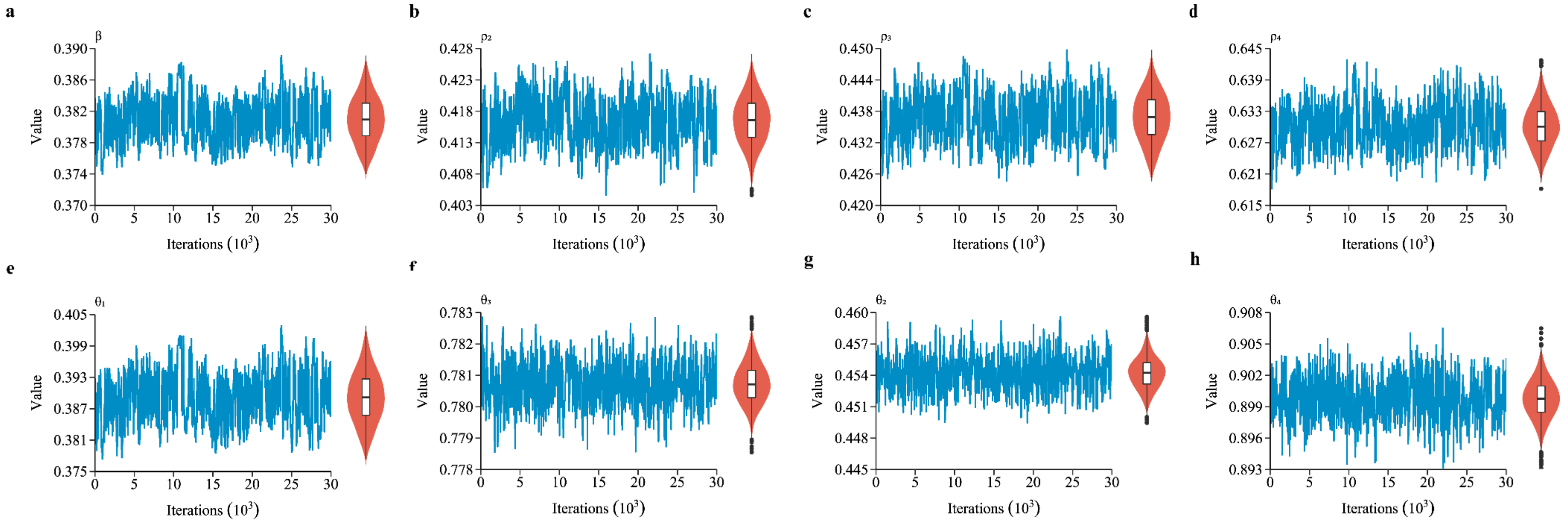

Figure S3. Trace plot of estimated parameters from the main analyses by MCMC and violin diagram of parameters. The blue line represents the trace plot of the parameters obtained by MCMC and the violin plot represents the distribution of the parameters estimates from 30,000 MCMC samples. In each violin plot, the thick line in the middle represents the median, the thick bar represents the interquartile range and the thin bar represents the minimum and the maximum.

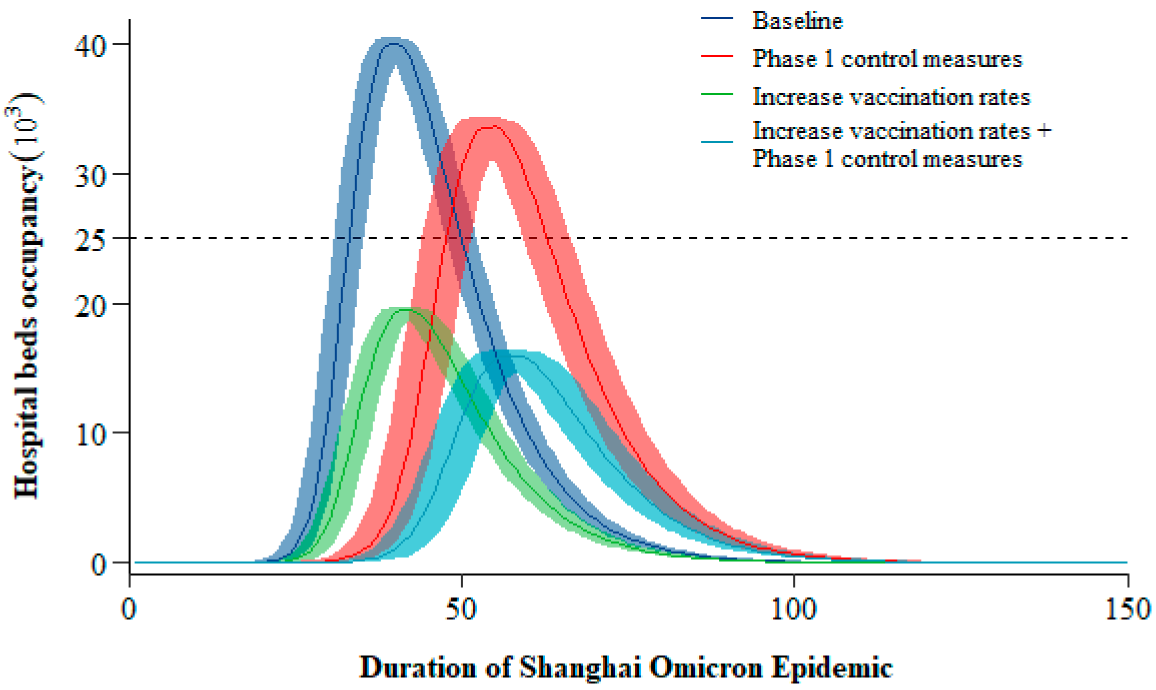

Figure S4. Projected demand and shortage of hospital beds under four different scenarios. Daily demand of hospital beds. Data are presented as median with 2.5% and 97.5% quantiles of 2,000 simulations. The scenarios included in legend are as follows: Blue indicates no additional control measures expect for basic public health measures under the existing vaccination coverage in Shanghai (Baseline). Red indicates the additional use of phase 1 control measures relative to the baseline scenario. Green indicates increased vaccination coverage relative to the baseline scenario. Light blue indicates increased vaccination coverage and the additional use of phase 1 control measures relative to the baseline scenario.

Reference

1. The Government of the Hong Kong Special Administrative Region, Hong Kong Vaccination Dashboard. Available online: https://www.covidvaccine.gov.hk/en/dashboard (accessed on September 25).
2. Zhang, J.; Klepac, P.; Read, J.M.; Rosello, A.; Wang, X.; Lai, S.; Li, M.; Song, Y.; Wei, Q.; Jiang, H.; et al. Patterns of human social contact and contact with animals in Shanghai, China. *Sci Rep* **2019**, *9*, 15141, doi:10.1038/s41598-019-51609-8.
3. Li, R.; Metcalf, C.J.E.; Stenseth, N.C.; Bjornstad, O.N. A general model for the demographic signatures of the transition from pandemic emergence to endemicity. *Sci Adv* **2021**, *7*, doi:10.1126/sciadv.abf9040.
4. Li, R.; Pei, S.; Chen, B.; Song, Y.; Zhang, T.; Yang, W.; Shaman, J. Substantial undocumented infection facilitates the rapid dissemination of novel coronavirus (SARS-CoV-2). *Science* **2020**, *368*, 489-493, doi:10.1126/science.abb3221.
5. Chen, X.; Yan, X.; Sun, K.; Zheng, N.; Sun, R.; Zhou, J.; Deng, X.; Zhuang, T.; Cai, J.; Zhang, J.; et al. Estimation of disease burden and clinical severity of COVID-19 caused by Omicron BA.2 in Shanghai, February-June 2022. *Emerg Microbes Infect* **2022**, 1-10, doi:10.1080/22221751.2022.2128435.
6. Cai, J.; Deng, X.; Yang, J.; Sun, K.; Liu, H.; Chen, Z.; Peng, C.; Chen, X.; Wu, Q.; Zou, J.; et al. Modeling transmission of SARS-CoV-2 Omicron in China. *Nat Med* **2022**, *28*, 1468-1475, doi:10.1038/s41591-022-01855-7.
7. Du, Z.W.; Liu, C.F.; Wang, L.; Bai, Y.; Lau, E.H.Y.; Wu, P.; Cowling, B.J. Shorter serial intervals and incubation periods in SARS-CoV-2 variants than the SARS-CoV-2 ancestral strain. *J Travel Med* **2022**, doi:10.1093/jtm/taac052.
